# Cancer diagnosed during pregnancy: a qualitative study of women’s psychosocial experiences during treatment and survivorship

**DOI:** 10.1007/s00520-026-10645-7

**Published:** 2026-04-23

**Authors:** Jenny Harris, Afrodita Marcu, Faith Gibson, Emma Ream, Karen Poole, Jane Stewart, Jo Armes

**Affiliations:** 1 School of Health Sciences, Faculty of Health and Medical Sciences, University of Surrey, Guildford, UK; 2 Centre for Outcomes and Experience Research in Children’s Health, Illness and Disability (ORCHID), Great Ormond Street Hospital, London, UK; 3 Clinical Trials and Statistics Unit, The Institute of Cancer Research, Sutton, UK

**Keywords:** Cancer survivorship, Pregnancy-associated cancer, Psychosocial support, Qualitative research, Decision-making

## Abstract

**Purpose:**

Cancer diagnosed during pregnancy presents unique challenges, requiring women to navigate treatment alongside pregnancy and early parenthood. While clinical aspects are well studied, the psychosocial impact on survivorship remains underexplored. This study examined the lived experiences of women diagnosed during pregnancy, focusing on emotional, psychological and practical challenges from diagnosis through survivorship.

**Methods:**

A qualitative study was conducted using interview data from 20 women in the UK diagnosed with cancer during pregnancy. Participants were recruited via Mummy’s Star, a charity supporting individuals affected by cancer in pregnancy. Interviews were thematically analysed using template analysis, focusing on decision-making, psychosocial burden and support needs.

**Results:**

Six inter-related themes were identified: (1) *managing cancer with uncertainty*, women reported distress due to delayed investigations and treatment adjustments during pregnancy; (2) *ethical decision-making*, emotionally charged choices around treatment, birth and feeding were made with limited or conflicting information; (3) *balancing cancer and its treatment with pregnancy and family life*, early parenting was disrupted; (4) *work disruption and financial strain*, treatment-related costs and lost income caused hardship; (5) *emotional impact of diagnosis and treatment*, including lasting psychological effects; and (6) *Coping and support*, guilt, fear of recurrence and unmet support needs persisted post-treatment.

**Conclusions:**

Women diagnosed with cancer in pregnancy face profound, long-term emotional and financial challenges. Fragmented care and inadequate support exacerbate these difficulties. Integrated multidisciplinary care is essential to improving survivorship.

**Supplementary information:**

The online version contains supplementary material available at 10.1007/s00520-026-10645-7.

## Introduction

Globally, an increasing number of women are diagnosed with cancer during pregnancy, a trend likely influenced by delayed parenthood in developed economies and the increased use of prenatal testing for foetal chromosomal abnormalities [1, 2]. While a growing body of research has examined the epidemiology and clinical management of cancer during pregnancy [3–6], including oncological, maternal and infant outcomes, relatively little attention has been given to the psychosocial impact of receiving a cancer diagnosis and navigating treatment and survivorship, alongside new parenthood, during this uniquely vulnerable time [7, 8].

The experience of cancer during pregnancy presents profound psychological and emotional challenges. Women often report high levels of distress, anxiety and isolation, exacerbated by the uncertainty surrounding treatment decisions, concerns about foetal health and the perceived lack of appropriate support networks [9–13].

Additionally these women frequently struggle to find peer support groups that feel relevant to their experiences and consequently endure isolation during treatment, and healthcare professionals (HCPs), despite their best efforts, may lack the experience or confidence to provide tailored psychosocial care [7, 14].

International research indicates that the psychological burden of a pregnancy-associated cancer diagnosis extends well beyond childbirth, with implications for long-term survivorship, maternal identity and family dynamics [9]. However, there remains a significant gap in understanding these experiences, particularly within the context of universal healthcare systems such as the UK’s National Health Service (NHS) [8] where access to psychosocial care in survivorship is managed by primary care.

Our recent UK-based qualitative study [15] examined women’s pathways to cancer diagnosis during pregnancy, revealing that symptoms were frequently misattributed to pregnancy-related changes, by both the women themselves and the HCPs they consulted. As a result, many women faced delays in diagnosis, requiring repeated visits to primary care before receiving specialist referrals. While the study focused on diagnostic pathways, participants also shared detailed accounts of their treatment experiences and survivorship concerns, underscoring the need for further exploration of these important phases.

Given the limited research on the post-diagnostic journey of women with pregnancy-associated cancer, we conducted this analysis as part of the pre-planned objective of a qualitative interview study [15]. The aim was to explore the psychosocial trajectory of women following their cancer diagnosis, throughout pregnancy and postpartum, including their experiences of treatment, support, decision-making and survivorship. By understanding these experiences, we can better inform service provision, improve patient-centred care and identify priorities for future research and practice.

## Method

### Design, procedures and sample recruitment

This study involved analysis of qualitative interview data collected to explore the experiences of 20 women who received a cancer diagnosis during or soon after pregnancy. Full methodological details of the study are reported elsewhere [15]. In summary, participants were recruited via Mummy’s Star, a charity dedicated to supporting women and families affected by cancer during pregnancy or within the first year postpartum. A study advertisement was shared on the charity’s online forum to facilitate recruitment.

Women were eligible to participate if they resided in the UK and had received a cancer diagnosis during pregnancy (or had sought help during pregnancy, but formal diagnosis was made postpartum) within the 4 years preceding recruitment, starting from January 2018. Women were excluded if they were diagnosed before January 2018, were currently pregnant at the time of the study or were less than 3 months postpartum. Sampling and data collection were guided by the principle of *information power* (guided by data depth, relevance and richness) [16].

Interviews were conducted online via Zoom between January and May 2022 by an experienced female social psychologist (AM), who was also a new mother with an academic interest in cancer early diagnosis. Interviews followed a semi-structured format guided by an interview schedule, allowing participants to share narrative accounts of their experiences. Interviews lasted between 36 and 66 min, were digitally recorded and were transcribed verbatim.

Prior to the interviews, AM gained informed consent and collected demographic, cancer and maternal history data. This pre-interview engagement helped familiarize participants with the researcher and ensured they understood the study’s purpose. Participants received reimbursement of £25 for their time.

The conception of this study was developed informed by an open academic, practice and public community engagement workshop held at the University of Surrey to identify priorities for psychosocial research around cancer and pregnancy with discussions and recommendation captured live by an artist (Supplement 1). Patient and public involvement was integral to the study design, with two women who had lived experience of cancer during pregnancy contributing to the development of study materials, including the participant information sheet and interview topic guide [15]. Ethical approval was obtained from the University of Surrey Ethics Committee (FHMS 20–21 199 EGA) and adhered to The Concordant to Support Research Integrity (UK).

### Analysis

We employed template analysis [17, 18]. This structured form of thematic analysis is widely used in qualitative psychology research, particularly when a priori themes have been established through prior analysis [17].

The analytical process began with data familiarisation, followed by preliminary coding to identify key themes. An initial coding template was developed iteratively and refined as additional data were analysed. Once finalised, this template was systematically applied to all transcripts using NVivo [19], ensuring a structured yet flexible analytical approach.

Template analysis balances inductive and deductive approaches [18] and accommodates various epistemological perspectives. For this study, we adopted a realist perspective [17], focusing on women’s experiences from diagnosis through treatment and into survivorship. Our research team included members with expertise in psychology, nursing and midwifery. The analysis began with JS (midwife), conducting an initial review of the dataset. The coding template was developed based on four key sources:

1. Prior knowledge of challenges faced by women diagnosed with cancer during pregnancy.
2. A scoping review and gap analysis [8].
3. Findings from the study on pathways to diagnosis [15].
4. Preliminary analysis of a subset of five transcripts (conducted by JH, JA and JS), selected to represent diverse cancer types and diagnostic timings (during pregnancy and postpartum).

The initial template was applied across the dataset by two authors (JS and JH independently), and coding discrepancies were identified through regular analytic meetings (JS, JH and JA) as well as additional meetings including AM (who conducted the interviews), resolved through discussion and negotiated consensus with reference to original transcripts. Final refinements were made to thematic category descriptors to reflect the emerging themes (JH and JA). This iterative approach ensured the analysis remained responsive to the dataset while maintaining a structured framework for interpretation. Member checking was not used in this study. While sometimes recommended in qualitative research, there is increasing methodological debate about its epistemological appropriateness and its contribution to rigour, as well as unintended consequences such as participant burden and distress, when studies involve sensitive experiences [20–22]. Given this debate, the topic area and the use of template analysis, rigour was instead ensured through independent coding, reflexive team-based analysis and iterative template refinement.

## Results

### Sample characteristics

Full details of the sample are described elsewhere [15]. The sample comprised 20 women, aged between 27 and 45 years (median age 35.5 years) (Supplement Tables 2 and 3). Their age at diagnosis ranged from 26 to 44 years (median 33.5 years). Most were married or cohabiting (*N* = 17, 85%). Fourteen (70%) were diagnosed during the coronavirus disease 2019 (COVID-19) pandemic, with the remaining being diagnosed before it. Seventeen participants (85%) received their diagnosis while pregnant, ranging from 5 to 35 weeks of pregnancy, while three (15%) were diagnosed 6–16 weeks postpartum after initially seeking help for symptoms during pregnancy. Ten participants (50%) gave birth at full term (i.e. > 37 weeks gestation). The remaining 10 gave birth preterm (following induction or C-section) after their cancer diagnosis (all between 33 and 36 weeks). Two-thirds were diagnosed with breast cancer (*N* = 13, 65%). Four women were diagnosed with advanced disease (20%).

### Themes

**Fig. 1:**
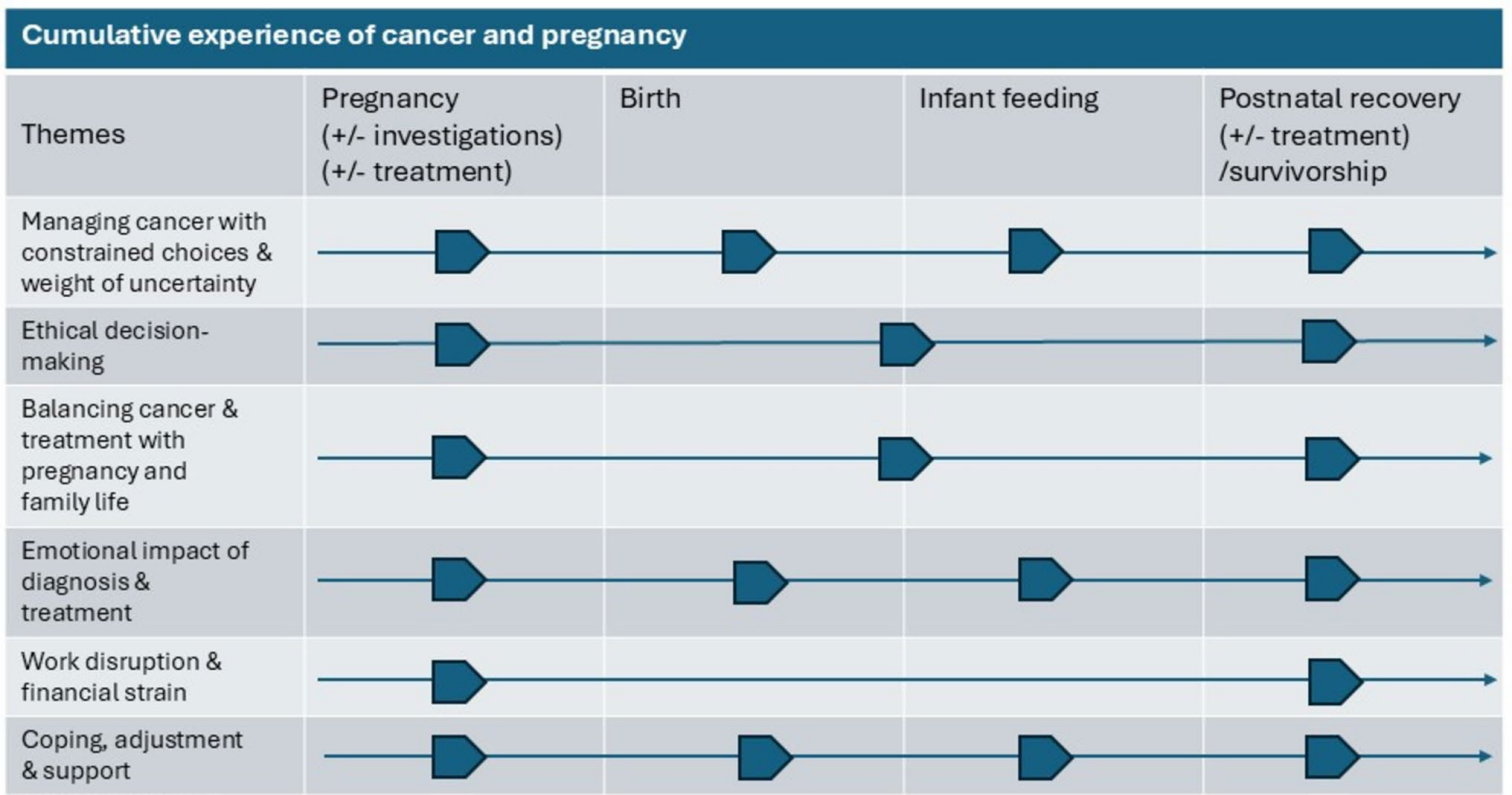
Cumulative experiences of cancer and pregnancy across key phases of the perinatal and survivorship pathway. The cumulative experience of cancer during pregnancy, mapped across four key phases (pregnancy, birth, infant feeding and postnatal recovery/survivorship, including ongoing treatment where applicable), is illustrated. Themes were overlapping, and the arrow symbol denotes points in the care trajectory where themes were most prominent, although themes commonly spanned multiple phases of pregnancy

Participants shared their experiences in a sequential fashion, meticulously recounting their pathway from treatment, childbirth and the aftermath, following their cancer diagnosis. Narratives were replete with intricate descriptions, and analysis produced six inter-related psychosocial themes, sometimes spanning the antenatal and postnatal phases (Fig. 1 and Supplement 4).

#### Managing cancer with constrained choices: the weight of uncertainty

Many women experienced the strain of facing limited diagnostic and treatment options during pregnancy. The inability to undergo standard investigations, staging and treatment often led to delays or altered care, leaving some with unanswered questions and heightening women’s fears about disease progression:

That’s the other thing, you are then being told all the things you can’t have. So, I couldn’t have the bone scan, I couldn’t have Herceptin, there was quite a lot of things they can’t do when you are pregnant. That’s always a bit of a worry […] because it was quite an aggressive cancer […] they wanted me to have the longer cycles of chemo […] Some people have, is it 9 or 8 cycles? I had 12 cycles because they wanted to be quite aggressive, and they weren’t going to be able to fit all the chemo in before they wanted the baby to be born. (P4; breast cancer)

Feelings of uncertainty and possible decisional regret were protracted for women who later discovered their disease was more advanced than initially expected:

I was diagnosed when I was 16 weeks, […] I didn’t realise some women have chemo and diagnostics while they are pregnant. But they just didn’t want to do that with me because they diagnosed via a biopsy. They said no treatment. […] maybe if they knew the full extent, I might have been offered treatment. But I’ll never know that. (P3; breast cancer)

These uncertainties sometimes had profound long-term consequences, particularly when a delayed diagnosis was associated with advanced disease. One woman with bowel cancer, whose symptoms were initially misattributed to pregnancy, was not diagnosed until her third trimester. By then, the priority was managing bowel obstruction, and standard investigations and cancer staging had to be postponed until after delivery. During survivorship, she reflected on the impact of delayed diagnosis:

By the time they took the tumour out […], and then they scanned me […] because they could now do a proper scan, because the baby wasn’t in there anymore, we found out that it had gone to my liver. [pause] Whether it was already going to go to my liver or not, you don’t really know. But if I had been diagnosed earlier in the pregnancy, I guess I could have had some […] So, I know that it is possible to have chemo during pregnancy. I probably would have done that. Then, that may have meant that the cancer would at least have been just contained in the colon and then wouldn’t have gone to my liver. It has now gone to my liver and my lungs. I have had two recurrences. The liver twice, I have had two liver ops, and then one lung operation. I have had a major operation every year since I got diagnosed. […] I think [chemotherapy] would have helped my long-term prognosis. (P10; bowel cancer)

#### Ethical decision-making processes

For many women, navigating treatment decisions during pregnancy was emotionally taxing. When they believed they had a choice, they faced the burden of weighing whether to proceed with investigations or treatments or to delay them, while balancing their own health, the well-being of their unborn child and, in some cases, the needs of their existing children.

One woman described the turmoil of deciding whether to proceed with surgery despite possible risks to her unborn child:

I had this real emotional rollercoaster […] Because obviously there is a risk of miscarriage during surgery under general anaesthetic, I had this real emotional rollercoaster of well if I don’t go ahead [I’d] … wait another seven, eight, possibly nine months […] I came to the conclusion of [name of child] is already here, [name of child] already knows me, I’m already [name of child] mummy, and obviously baby didn’t at the time, so I just kind of had to try and break that emotional attachment to try and be pragmatic in making that decision. (P5; thyroid cancer)

For some, the emotional weight of these decisions was compounded by the need to advocate for themselves in conversations with healthcare professionals, particularly when they felt their preferences were not being fully acknowledged. Some women found themselves needing to assert their preferences against HCPs’ recommendations:

It was just a feeling within me, when I got to 35 weeks, I just had this really strong feeling and I said […] I’ve done everything […] I’m continually in this hospital having scans. I’m glad [infant]’s grown to a good size. The obstetrician was quite reluctant, but then […] [the obstetrician] agreed […] So I was induced […] emotionally I felt like… I think it had got to a point where I’d known of this [cancer] in me for however many months, four months, and I just wanted to get on with the treatment because all I cared about was being there and trying to have some of my maternity leave to just enjoy my daughter […] And I didn’t want my cancer to spread any more than it already had either […] it was a relief to have her. It was a real strain carrying her in my pregnancy and all that was going back and forth in my mind. (P3; breast cancer)

Many actively sought information to support their decisions, yet some struggled with guilt and decisional regret:

I was able to get some of the information about the risks with things like early labour, stillbirth rates. I remember discussing all that, but for quite a long time it was “what am I doing?” I just felt so guilty […] my need for a child was such that I was prepared to put this baby through […] all this chemo, and who knows what the outcome of her would be, just because I felt quite selfish, what effect could this have on her? (P4; breast cancer)

For some, the absence of information and not being offered choices by clinicians reinforced a sense of lost agency. One woman had to independently research reconstruction options, as she had not been informed about them:

I had the option whether I wanted to go ahead or not, but I didn’t have any options in terms of what I’m having… I chose to have the implant put in which I’d already done my research and asked for, but it wasn’t an option that was given to me, I’d sort of looked into that and requested it. (P11; breast cancer)

Beyond treatment, some women felt disempowered in other aspects of their care, such as infant feeding:

They basically said you can’t [breastfeed]. So yes, that was very sad for me because I loved breastfeeding […] And in all honesty, that was the thing I found hardest in the whole diagnosis. I could sit and I could talk to anyone about it and the only thing that ever made me cry was that I wouldn’t be able to breastfeed my second baby, because it was so important to me to be able to do that […] when the bottle making machine turned up at the door I burst into tears and stuff like that. It was very strangely the hardest thing I found about the whole experience […]. (P14; breast cancer)

This same woman, however, was able to negotiate with her oncology team, demonstrating how persistence sometimes influenced clinical decisions:

I think because I kept breaking down into tears anytime anyone talked about breastfeeding, or every time I brought up breastfeeding. I think my team realised how important it was to me and my oncologist tried to give me as long as possible afterwards. So, [the oncologist] initially maybe said two weeks, which I thought was just a joke because by two it’s just that’s no time at all, is it? Then [the oncologist] kind of said maybe two to three weeks and then I kept negotiating with [the oncologist] a little bit more and, yes, I think I ended up being able to breastfeed for four weeks. (P14; breast cancer)

#### Balancing cancer and its treatment with pregnancy and family life

A common theme was the ongoing challenge of managing the dual demands of cancer care and motherhood, often with lasting physical and emotional consequences. The transition into motherhood, typically a time of bonding and recovery, was disrupted by the necessity of prioritising cancer treatment. The relentless juggling act of balancing medical appointments, treatment side effects, postnatal recovery and childcare often left women physically and emotionally drained. The enduring impact of these experiences shaped not only their recovery but also their ongoing journey as both cancer survivors and mothers:

I found it really exhausting because I was still trying to look after my other two children, do things like the school run. I found it exhausting and sometimes really overwhelming because the amount of appointments I’d have. Some days I might be seeing my haematologist one day at one hospital in (one location), the next day at a different hospital in (another location) for a scan, I used to have scans every two weeks, then I might be seeing the midwife the next day, then I might be going to my GP’s the day after that to have my pre chemo bloods done and then I might be, on the Friday, going and having the chemo. And it was just exhausting. (P1; Hodgkin’s lymphoma)

Logistical challenges were amplified for a woman needing to manage the specific requirements of thyroid treatment:

[Due to treatments involving radioactive iodine] I would have had to stay away from home for another four days, so I had the issue of logistics of where would I stay… the friends that I do have all have young children as well, so that was another real mental headache. (P5; thyroid)

These logistical and emotional challenges did not end with childbirth. For many, treatment extended into or commenced in the postpartum period, compounding the sense of exhaustion and feeling overwhelmed.

There was a lot going on at all times. It was really quite difficult to manage. I don’t think I actually […] missed any appointments, […] but it would have very easily been done […] sometimes it was just so overwhelming, the amount of appointments. (P14; breast)

The simultaneous challenges of cancer and pregnancy compounded the usual fatigue associated with both experiences. Women described an overwhelming sense of exhaustion, which extended into their postpartum recovery as they juggled motherhood and ongoing treatment which one woman described as “gruelling” (P13; breast cancer).

The need to prioritise cancer treatment over new motherhood often meant that women were unable to fully experience early bonding moments with their babies. For some, the transition from pregnancy to intensive cancer treatment was abrupt, leaving little time to recover physically or emotionally.

I felt like when people talk about the baby bubble and you get to spend those first few weeks just at home and cuddling and having a nice time, breastfeeding, just being close and bonding with your baby, especially the very first time that I had to get up and leave the house and go for chemotherapy, it was just heart-breaking. Yes, I just felt like it was a very abrupt end to almost […] It almost felt like it was like, right, well that’s that then, that’s finished. (P14; breast cancer)

For some, the necessity of immediate treatment further complicated birth and recovery experiences, disrupting their ability to care for their newborns:

Then, when the baby came, we were just happy that [infant] is okay, and then [infant] goes to NICU [due to planned pre-term delivery at the same time as her surgery] […] Going up to visit the baby was really hard, because I had to be taken in a wheelchair by someone else. I couldn’t get there myself. I was finding it hard to express milk. I wasn’t really eating much, so the milk wasn’t really coming. I was just worried that I wasn’t going to visit enough, and everyone would think I was an awful mother, because I was never up there. All the other mums would sit by their incubator all day, but I would go there for about ten minutes, and I just couldn’t sit there anymore, because I was so ill. (P10; bowel cancer)

The intersection of cancer treatment and pregnancy often resulted in medical complications that intensified the already demanding experience. Some women experienced unexpected surgical complications, further prolonging their recovery:

My wound was leaking. I thought it was just breast milk from the other breast, but it was actually my wound was leaking I think it was just some excess fluid or something with the pressure of labour. (P9; breast cancer)

For one woman, a caesarean section led to additional complications that significantly impacted recovery and mobility, adding yet another layer of difficulty to an already overwhelming situation:

When they did the C-section, […] they also cut my bladder, and I got a wound infection from the C-section So, I had lots of complications […] on top of everything else. I had a catheter for six weeks […] while my bladder healed. It was a complete nightmare. A nightmare month in hospital […] The bladder injury […] just complicated things loads because it just added another thing to the list of things that were already bad. It made me much less mobile, and it added much more hospital appointments and […] scans […] I had urine coming out of my C-section scar, pouring out of me. Then they let us both out […] I was at home for about a week, and then I went back [in for] the tumour surgery. (P10; bowel cancer)

#### Work disruption and financial strain

As the women were of working age, many described the strain of managing cancer, pregnancy and work simultaneously. Cancer often disrupted women’s plans to work during pregnancy or return to work after maternity leave, forcing difficult choices about priorities and capacity:

I couldn’t cope with work. I didn’t have the mental headspace for being pregnant, having cancer and working. I obviously have done being pregnant and working. I think I could have done cancer and working to the point… but I couldn’t do them all…Keeping a track of what the doctors were saying, medicine, where to be, appointments. (P4; breast cancer)

These disruptions to employment were often accompanied by significant financial strain particularly as frequent appointments, travel and unpaid leave compounded existing expenses:

It has a big financial implication […] you’re having to travel a lot to these appointments […] things like petrol (gas), diesel, and then having to pay to park at the hospital as well […] Because you are at higher risk from the cancer, you then need to see your obstetrician […] more often[…] so you are paying quite a lot of money to be in a car park and obviously I didn’t work then, I was off sick from work […] You’ve got enough to worry about without having to worry about money. Sometimes I had to borrow money from my mum to put petrol in my car to go to my hospital appointments. I didn’t have any money. (P1; Hodgkin’s lymphoma)

These financial challenges could be compounded for those who already had children:

I just needed some support. I did need a babysitter […] if […] you have a toddler and you have a baby but because of your circumstance you can’t physically look after them. We got out a loan and we spent loads of money on childcare because I couldn’t look after them sometimes. That was really difficult and we’ve only just paid it off three years later. (P3; breast cancer)

Some had partners who were able to take time off work to support them and handle family responsibilities. Nevertheless, this could result in greater financial strain:

Obviously with my partner not being able to work because he was looking after me and the baby and then me not working and my maternity pay obviously runs out [talking about receiving free counselling]. I don’t think I would have sought that sort of help if I’d had to pay for I […] I would have thought but there’s so much I need to [buy] look after my daughter, the money should really go to that. (P16; breast cancer)

In response to these challenges, some women turned to cancer charities for practical support and financial guidance:

I have been in contact with [charity named], but that was more helping out with finances, if you are ill and you can’t work […] I went and saw someone who helps you apply for what benefits you can get, and what to do, and stuff like that. That was good […] help with life, benefits, things like that. (P10; bowel cancer)

#### Emotional impact of diagnosis and treatment

The immediate aftermath of diagnosis was often marked by overwhelming feelings of threat, fear and emotional paralysis:

I was just in shock […] I just felt so locked in. I didn’t do anything. All I remember of that period is sitting in the beanbag chair watching telly, nothing else, in this empty room. (P7; breast cancer with secondary disease)

However, for some, the magnitude of these feelings were delayed as they initially attempted to cope by continuing their regular routines:

When I was told, “You’ve got cancer,” […] it didn’t sink in, and I wanted to know that my baby would be safe, that was my main concern really, and I was just in absolute shock to be honest with you, and it took quite a long time. I put on this brave face, and I think it was like a few weeks after, I just couldn’t stop crying and I had to call my husband. I parked outside his work and he just said, “I did think you were taking it very well”. And I just don’t think it had sunk in because I was looking after my son. I was working four days a week, I was carrying a baby, busy times. And this was just completely out of the blue, unexpected […] I was devastated, confused […] and searching for answers. (P3; breast cancer)

As the shock of diagnosis gave way to daily realities, many women described navigating intense emotional contradictions of joy and fear, and hope and despair, often within the same moment. The same women described regaining emotional control by compartmentalising these contradictory thoughts and emotions:

It’s a completely conflicting stage of your life; you should be happy, and people want to congratulate you. And actually, at work I decided not to tell anyone except my immediate teams. There were three people that knew and everyone else just kept saying, “Oh you must be so excited,” and I was, but in the back of my mind I knew the diagnosis and it was a real battle in my mind […] I don’t really know, looking back, how I kept all those feelings under control […] It was a real rollercoaster of emotions […] and as we did with my son, we opted to find out the sex of the baby and I was overjoyed it was a girl because that’s what we wanted, one of each, so that helped me feel more connected and gave me that real drive to just keep going and keep going. (P3; breast cancer)

Many accounts detailed efforts to retain a sense of normality during a disrupted antenatal and postnatal period, highlighting the emotional toll of guilt and social withdrawal:

For the first couple of weeks, it was a nice positive thing, like having a baby and it was something to look forward to and then you start (more investigations) […] got the CT scan and then it was back to all about the cancer. Then you are starting treatment. I feel like I pushed myself so much to still do things, like still make sure I was doing my fair share with [son], even probably when I shouldn’t have, I should have just been taking my time. But I feel like I did keep pushing myself. I think there was a lot of guilt there. I sometimes I couldn’t look after him, I couldn’t look after both my kids and that was quite hard, I was relying on my mum and dad a lot and my husband and I felt like a bad mum […] It’s hard because you know it’s not your fault, but you still feel that guilt. Some days I just wanted to hide in my room and just hide under the duvet and not come out. I did [laughs]. But yes. It was hard. (P9; breast cancer)

Women also showed concern about the emotional impact of their cancer their partners and extended family members, frequently experiencing guilt for the distress caused to their loved ones:

Massive fear…… So how are we going to tell family? Guilt that I was bringing it on the family as a whole, which I know is utterly ridiculous, but at the time I did feel really guilty that, at a time when it should have been a time of joy for the family in having my daughter – [infant] is the first grandchild on both sides – so going from the joy of having a [n] week-old baby to having lockdown, where we didn’t know whether grandparents would be able to see her, to not only having that but then the cancer diagnosis. I felt really guilty that I was adding more stress and worry to everybody’s plates, not just ours. (P18; breast cancer)

Some women found themselves supporting their partners and families while simultaneously managing their own adjustment. One woman, experiencing a recurrence, adopted a problem-focused approach:

[My Partner] was really upset, crying, and I just turned round and said, “We’ve done this. We can do it again”. I just said [to her partner], “Right, tell me what we need to do, and we’ll take each week as it comes”. (P16; breast cancer with bone metastases)

### Coping, adjustment and support

Many described how cancer had permanently changed them, leading to long-term psychosocial adjustment challenges years later, as described by two women both with breast cancer:

I used to be quite outgoing, quite a social person. I’m finding it really difficult to go out now, quite anxious about seeing people […] lacking in confidence in myself, not just appearance but everything. (P9; breast cancer)

I read something recently that said being diagnosed with cancer is like having a gun placed to the back of your head. It’s always there, you know it’s always there […] it’s just learning to live with it always being there […] I find I need to manage one day at a time, one week at a time. (P4; breast cancer)

For some diagnosed during COVID-19 restrictions, lingering frustrations about care experiences later exacerbated their fears of recurrence:

I’m angry with myself […] and I’m angry with them that it took so long […] With my birth as well, I was angry that they didn’t know about my situation and they didn’t have the compassion for my husband to [due to Covid …] stay with me. (P9; breast cancer)

Women described varied experiences with support, ranging from deep isolation to resilience fostered by social networks:

It was frustrating because I’d found the [names support group] very helpful, but you only get to go to that once. It’s run every year but once you’ve been once that’s it […] I always felt very alone and very isolated. That didn’t help my mental health. I had a massive crash mentally afterwards because just, well, everything got on top, I suppose. (P4; breast cancer)

Specialist psychological support was often seen as more beneficial later in their survivorship journey, rather than during treatment:

I’ve not found counselling very helpful, to be honest. […] At that time, it wasn’t as easy to talk about it when you are going through the throes of treatment […] I’m considering therapy now […] but just I think maybe because all the dust has settled you start to really appreciate all the complexities of what you are feeling. But during (treatment) […] I don’t think I had the headspace for it and the counselling […] I think I was just too busy just surviving it. (P15; bowel cancer)

Some sought professional help for their mental health, while others recognised the importance of self-care and maintaining well-being during survivorship:

I did go for counselling after my active treatment finished, just because I was aware that I had been through a lot […] I knew that I needed to make sure I was alright emotionally and mentally […] I needed to make sure I had time to reflect and accept what had happened to me as well […] I know that at certain times I might need to go back into that […] just to make sure I’m keeping myself well mentally as well as physically. (P18; breast cancer)

## Discussion

This study provides important contributions to the research into the complex experiences of women navigating cancer survivorship when the diagnosis coincided with pregnancy. Although fortunately an uncommon diagnosis, incidence is increasing, and so it is important that cancer professionals are aware of the issues faced by these women and families in survivorship [14, 23] in order to support shared decision-making about treatment [24] and to provide support at the time when it is needed.

Our findings highlight the tension between autonomy and constraint in decision-making, where women’s choices about treatments, investigations and parenting decisions such as breastfeeding were frequently shaped or limited by external factors related to their cancer. While some women were able to negotiate aspects of their care, others felt their options were restricted or imposed, contributing to feelings of uncertainty, guilt and powerlessness. These high-stakes decisions, sometimes made contrary to medical advice or in the absence of clear guidance, placed considerable emotional strain on women already managing the dual challenges of cancer and pregnancy.

This aligns with the wider literature on decisional regret in oncology, which shows that constrained or unsupported decision-making is associated with lasting psychological distress, including anxiety, guilt and reduced quality of life [25, 26]. Decisional regret is particularly pronounced when patients feel they have received insufficient information or were pressured into choices that conflicted with their personal values [27]. Similarly, in the context of maternity and early motherhood, emerging research suggests that when women’s decisions during pregnancy and childbirth are constrained by external factors, this can contribute to sense of lost autonomy and subsequent regrets [28].

Our findings suggest that for pregnant women with cancer, these dynamics may be further amplified by the simultaneous need to consider foetal well-being, compounding the emotional complexity of care decisions and increasing potential for long-term psychological impact.

Studies have also reported that many women feel overwhelmed by the need to balance cancer treatment with pregnancy-related concerns, contributing to emotional distress, guilt and confusion about how to prioritise care [7, 8, 11, 29, 30]. Similar to our study, previous work has shown that the care of these women may be fragmented across multiple specialties with variable communication, contributing to feelings of being unsupported or misunderstood by their healthcare teams [23, 31].

The emotional disruptions reported by the women, with psychological stress emerging as a key theme in their journey, are consistent with those of other qualitative studies on cancer and pregnancy, which have similarly highlighted the emotional and psychological challenges faced by women in this context [7, 8, 13, 32].

Our findings align with the ‘burden of treatment’ theory, which describes the workload imposed on patients by healthcare systems and their capacity to manage that burden [33]. In our study, this burden was compounded by the unique challenge of navigating two life-changing events simultaneously: cancer and pregnancy. For many, this duality created not only competing demands, managing intensive medical care, adapting to the pregnancy, and in many cases, continuing caregiving and work responsibilities [34, 35], but also conditions of cumulative stress that may be experienced as traumatic. Several women described being in survival mode, focused on simply getting through the experience day by day. In this context, the act of enduring, rather than reflecting, became the priority. This response is consistent with trauma theory [36, 37], which recognises how chronic threat, uncertainty and loss of control can lead to psychological trauma, even in the absence of a single acute event. The cumulative impact of delayed emotional response, sustained stress and role overload may explain the longer-term adjustment difficulties some women described in survivorship and suggests the potential value of adopting a trauma-informed approach [37, 38] to post-treatment care. Such an approach emphasises safety, empowerment and understanding of trauma’s impact on physical and emotional well-being [39, 40] and may help address unmet psychological needs in this population [8, 41]. This warrants further research.

Financial precarity emerged as a consistent concern in our study, reflecting findings from the cancer survivorship research [42–44] and maternity and early motherhood literature [45]. This financial strain is particularly concerning given its established association with poorer mental health outcomes [46] and late effects [47]. However, the economic impact of cancer during pregnancy remains under-researched but it is likely that being diagnosed with cancer during pregnancy worsens this.

Women of childbearing age are often in the early or mid-stages of building their careers, and a diagnosis of cancer during this life phase may have lasting consequences for income, job security and long-term career progression. The economic burden of undergoing cancer treatment while pregnant, including costs associated with frequent hospital visits, treatment-related expenses and the potential loss of earnings due to reduced capacity to work, likely compounds the overall challenges of managing pregnancy-associated cancer.

In our study, these pressures were a recurrent theme, pointing to a significant gap in the literature around how best to support women with cancer during pregnancy, particularly in terms of financial guidance, workplace rights and reintegration into employment following treatment.

Maternal guilt [48], previously described in the context of high-risk pregnancies [49], was also intensified in the context of cancer and may be an issue that cancer clinicians working with these women are unaware of. Concerns about the health of their baby, their ability to manage treatment, ability to care and live up to their own expectations for existing children or their maternal role contributed to deep, long-lasting feelings of guilt. These emotional challenges can be traumatising and may have a lasting impact on women’s psychological well-being, potentially shaping their long-term adjustment and recovery [49]. Our findings emphasise the need for integrated psychological support, as many women actively sought out personalised care, often finding it difficult to find appropriate resources.

Although this study focused on women’s experiences, participants frequently described the emotional and practical impact of their diagnosis on partners and family members, highlighting the interdependent nature of distress within couples. Emerging evidence suggests that partners of women diagnosed with cancer during pregnancy experience significant psychosocial strain, including role changes, emotional burden and responsibility for caregiving and advocacy, with partner coping closely linked to maternal adjustment [9, 50]. While partner perspectives were beyond the scope of the present study, these findings underscore the importance of dyadic and family-oriented approaches to survivorship care. Interventions that acknowledge the couple or family as a unit, rather than focusing solely on the birthing partner diagnosed with cancer, may better support long-term psychosocial adjustment for both women and their partners [51].

Routine access to counselling and emotional support should be considered an essential component of care for women affected by pregnancy and cancer, given the profound and enduring psychological effects reported by participants in our study and others [2, 5, 13, 52]. However, the timing and format of such support warrant careful consideration as wider research suggests uptake of support can be low even when distress is high [53]. Our findings and existing literature suggest women are often in ‘survival mode’ during diagnosis, treatment and early motherhood; emotionally overwhelmed and focused on immediate tasks. In this context, women have limited capacity or headspace to engage and benefit from psychological interventions when they are offered in the immediate post-diagnosis or postnatal period. This reduced capacity can be understood through trauma theory and cognitive load frameworks, which propose that sustained threat, uncertainty and competing demands constrain emotional processing and therapeutic engagement [54]. During a period of high cognitive and emotional load, women may prioritise survival-focused tasks leaving little space for emotionally focused support [54]. These findings highlight the importance of recognising windows of capacity for psychosocial care [54] and suggest that flexible, longitudinal support pathways [55], rather than single time-point interventions, may be better aligned with women’s evolving needs across treatment and survivorship.

Furthermore, our study resonates with the broader body of research on maternal guilt and shame [48, 56]. Studies on women facing high-risk pregnancies [57], including those with cancer, consistently report feelings of guilt related to their health, treatment choices and maternal role. Our study further demonstrated that in the context of pregnancy and cancer, this guilt is compounded by concerns about the potential impact of cancer treatment on the foetus and the difficulty of managing both the emotional and physical demands of pregnancy alongside cancer treatment. Our findings are consistent with those of other studies on cancer and pregnancy, which have similarly highlighted a sense of isolation and uncertainty [30, 58, 59], as these women struggle to make decisions about their health and the health of their unborn child.

Rather than a one-size-fits-all approach, psychological support should be trauma-informed, flexible, ongoing and tailored to individual readiness. As described by participants, a diagnosis of cancer during pregnancy involves sustained threat, loss of control, uncertainty and competing demands, which may constrain capacity to engage. Trauma-informed care therefore provides a useful framework for understanding both psychological need and variability in engagement. Trauma-informed approaches emphasise safety, trust, collaboration and choice, and recognise how trauma responses can shape interaction with healthcare [38, 60]. In practice, this may include validating emotional responses, providing clear and consistent information wherever possible, minimising re-traumatisation associated with fragmented and siloed care. Recent clinical guidance supports routine joint oncology-obstetrics integrated multidisciplinary approaches for women diagnosed with cancer during pregnancy, also including foetal and neonatal specialists [61–63]. Further research is needed to understand how such models can be effectively implemented across different healthcare systems. Our research suggests that designated care navigators may potentially help reduce fragmenting care experiences for women, though this warrants further investigation. Integrated, longitudinal psychosocial pathways across oncology and maternity services could help ensure that support is available and accessible at time when women are most able to engage with it. Some women may particularly benefit once the intensity of treatment and early caregiving has eased, though individual needs and preferences are likely to vary considerably [59, 64]. Based on participants’ accounts and consistent with emerging international guidance [61–63], we summarise preliminary practice implications for cancer, maternity and wider healthcare teams in Supplement 5.

## Limitations and strengths

This study has some limitations. The sample size was relatively small and self-selecting, which may limit the transferability of the findings. However, given the aim of the study, the shared experience of participants (cancer diagnosis during or shortly after pregnancy) and the richness of data generated, our sample size was considered sufficient to support in-depth qualitative analysis based on the principle of information power [16]. Additionally, the study relied on retrospective accounts of participants’ experiences, which may be subject to recall bias. However, this was countered by the detailed description participants provided, including dates of diagnosis and other significant events. While the lack of cultural diversity in the sample may limit the applicability of findings to broader populations, the study did include geographic diversity across the UK and captured a range of experiences. There was also diversity in the quality of care received, some women were dissatisfied with their oncologist or obstetrician, while others reported positive experiences and felt well supported. Furthermore, the cross-sectional nature of the study limits our ability to qualitatively examine long-term psychological outcomes or the evolution of women’s experiences over time.

Despite these limitations, the strengths of this study lie in its novel contributions to understanding the psychological, emotional and financial burdens faced by women with pregnancy and cancer, an underexplored area in existing literature. By focusing on participants lived experiences, this study provides valuable insights that can inform future research and guide improvements in healthcare delivery for this population.

## Conclusion and recommendations

This study illuminates the significant emotional, psychological and financial challenges experienced by those affected by pregnancy and cancer, often exacerbated by fragmented care pathways. Healthcare systems need to prioritise the integration of multidisciplinary care teams that address not only the medical aspects of treatment but also the psychological, financial and social dimensions of these women’s experiences. Preliminary implications for survivors and families are summarised in Supplement 6. Future research should explore long-term psychological outcomes for women and families [65] and investigate interventions to improve care integration and support services. Enhancing survivorship care for women and families is key for improving quality of life both during and after treatment.

## Supplementary information

Below is the link to the electronic supplementary material.

ESM 1 (DOCX 21.3 KB)

ESM 2 (DOCX 606 KB)

## Data Availability

The research data are not shared because they contain sensitive and potentially identifying information.
