# Supplementary material for: Cancer diagnosed during pregnancy: a qualitative study of women’s psychosocial experiences during treatment and survivorship
Source: Support Care Cancer. 2026 Apr 23;34(5):458. doi: 10.1007/s00520-026-10645-7 (PMC13102820; doi:10.1007/s00520-026-10645-7)
Supplement: Supplementary file 1 — (DOCX 21.3 KB) [file 520_2026_10645_MOESM1_ESM.docx]

**Box S3. COREQ 32-item Checklist**

Authors: Jenny Harris, Afrodita Marcu, Faith Gibson, Emma Ream, Karen Poole, Jane Stewart, Jo Armes

| **Number** | **Item** | **Description** |
| --- | --- | --- |
| **Domain 1: Research team and reflexivity** | | |
| *Personal characteristics* | | |
| 1. | Interviewer | AM |
| 2. | Researcher credentials | All authors have PhDs |
| 3. | Occupation | Describe all of us |
| 4. | Gender | All authors are female. |
| 5. | Experience and training | All authors are active researchers in cancer early diagnosis, supportive cancer care, complex interventions, and midwifery. Each author received qualitative research training during their doctoral studies and has extensive experience in conducting qualitative research. |
| *Relationship with participants* | | |
| 6. | Relationship established | The study researchers had no prior relationship with participants before the commencement of the study. Participants were recruited via an online forum hosted by the charity Mummy’s Star (see page 4). They were informed that the study aimed to explore the experience of receiving a cancer diagnosis during or shortly after pregnancy. |
| 7. | Participants’ knowledge of the interviewer | None of the participants had any prior contact or existing relationship with the interviewer. |
| 8. | Interviewer characteristics | The research team brings longstanding expertise in examining how individuals make sense of potential cancer symptoms and navigate a diagnosis of cancer during pregnancy. The positionality of the interviewer is discussed on page 5. |
| **Domain 2: Study design** | | |
| *Theoretical framework* | | |
| 9. | Methodological orientation and theory | Template analysis, page 3-4. |
| *Participant selection* | | |
| 10. | Sampling | Sampling described page 2-3. |
| 11. | Method of approach | Online, page 2-3. |
| 12. | Sample size | 20 |
| 13. | Non-participation | Twenty-six women responded to the study advert. Two were ineligible as they were currently pregnant, and four did not complete consent procedures or confirm interview dates. The final sample included 20 women (see page 5). |
| *Setting* | | |
| 14. | Setting of data collection | The interviews were conducted online, page 2-3. |
| 15. | Presence of non-participants | No non-participants were present. |
| 16. | Description of sample | Provide page 5 and Supplement Table 2-3 |
| *Data collection* | | |
| 17. | Interview guide | Provided in prior publication. |
| 18. | Repeat interviews | n/a |
| 19. | Audio/visual recording | Interviews were audio-recorded and transcribed verbatim, page 5 |
| 20. | Field notes | n/a |
| 21. | Duration | Individual interviews lasted 36 to 66 minutes. |
| 22. | Data saturation | Our study was informed by information power, page 3 |
| 23. | Transcripts returned | No transcripts were returned to the participants. |
| **Domain 3: Analysis and ﬁndings** | | |
| *Data analysis* | | |
| 24. | Number of data coders | Described in full on page 4. |
| 25. | Description of the coding tree | n/a |
| 26. | Derivation of themes | Derived from data and applied to all transcripts. |
| 27. | Software | NVivo |
| 28. | Participant checking | We did not employ participant checking. |
| *Reporting* | | |
| 29. | Quotations presented | Indicative quotes are included in the manuscript under each theme and in Supplement 4. |
| 30. | Data and findings consistent | Yes. |
| 31. | Clarity of major themes | Yes, Figure 1. |
| 32. | Clarity of minor themes | Yes, Supplement 4. |
