# Supplementary material for: Cancer diagnosed during pregnancy: a qualitative study of women’s psychosocial experiences during treatment and survivorship
Source: Support Care Cancer. 2026 Apr 23;34(5):458. doi: 10.1007/s00520-026-10645-7 (PMC13102820; doi:10.1007/s00520-026-10645-7)
Supplement: Supplementary file 2 — (DOCX 606 KB) [file 520_2026_10645_MOESM2_ESM.docx]

**Supplement 1.** A selection of visual representations of priorities for education and research that informed this study. An artist, patients and the public were involved in at an online international priority setting workshop hosted by the Institute of Advanced Studies at the University of Surrey (July 2021). This event was attended by 60 delegates from nine different countries, including women and partners with lived experience, oncology and maternity healthcare professionals (HCP) and support staff, academic researchers and educators and representatives from several charities. The workshop gained consensus on the priorities for future research into the psychosocial well-being and support for women diagnosed with cancer and their families. The discussions and recommendations were captured live by an artist (Katie Chappell, www. katiechappell.com).


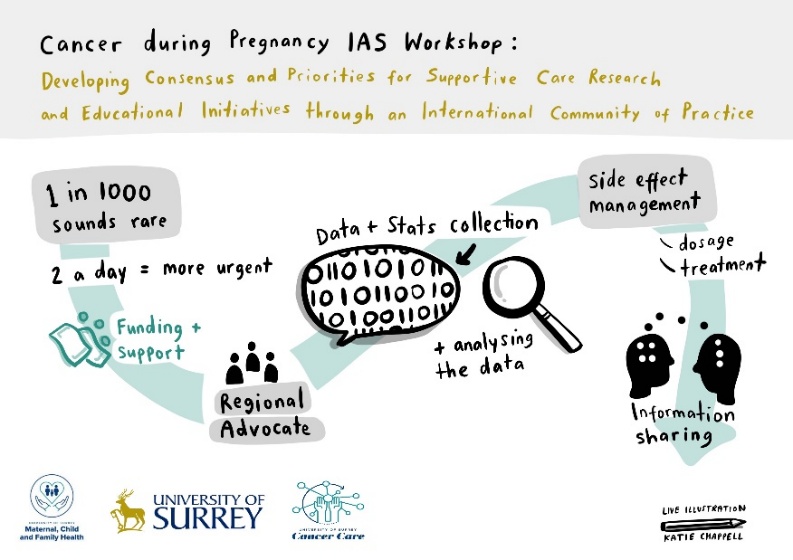

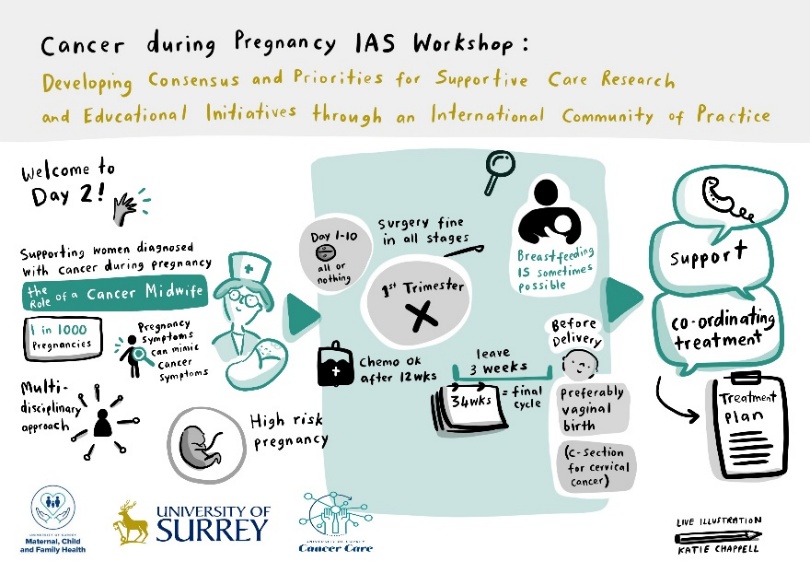


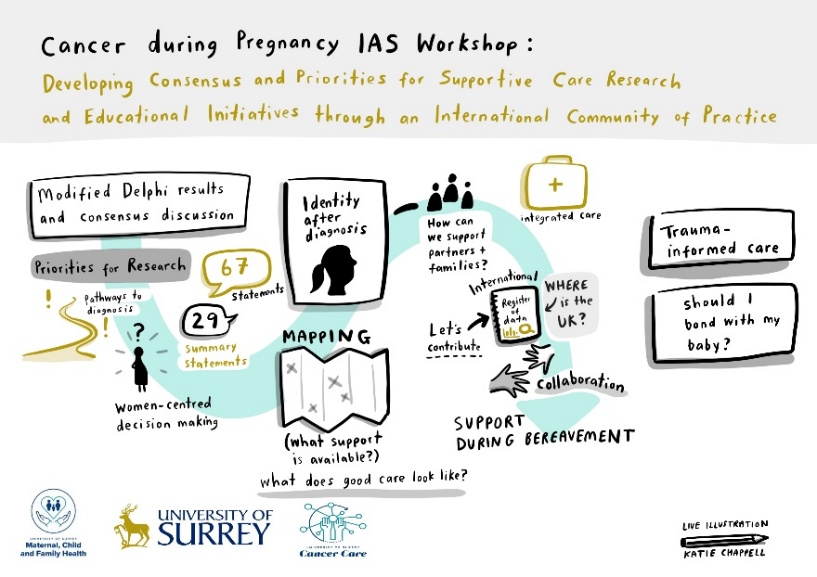

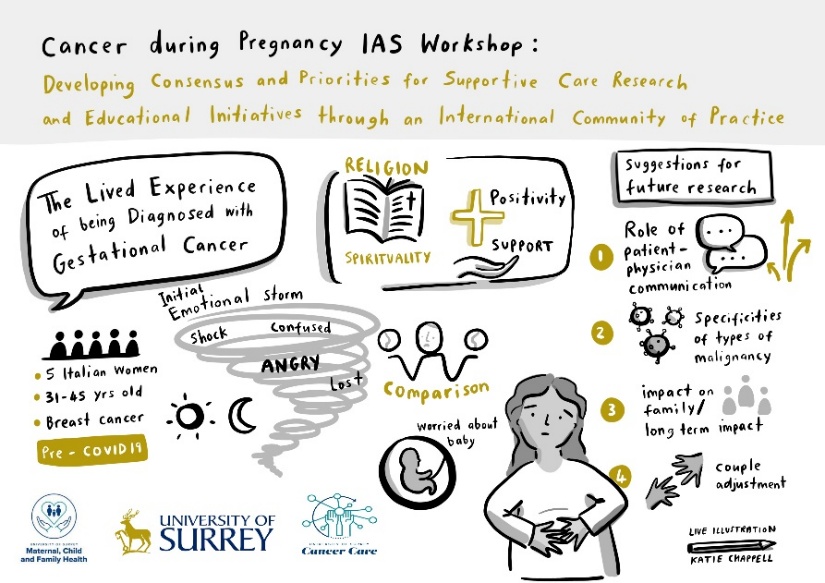


| **Supplement Table 2. Pregnancy-Associated Cancer: participant summary (N=20)** | | | | |
| --- | --- | --- | --- | --- |
| **Section** | **Characteristic** | **Category** | **n** | **%** |
| Demographics | Age Band | Under 30 | 3 | 15 |
|  |  | 30-34 | 5 | 25 |
|  |  | 35-39 | 8 | 40 |
|  |  | 40+ | 4 | 20 |
|  | Gestation at Diagnosis | First Trimester (5-13 weeks) | 2 | 10 |
|  |  | Second Trimester (14-27 weeks) | 7 | 35 |
|  |  | Third Trimester (28-35 weeks) | 8 | 40 |
|  |  | Diagnosis postpartum | 3 | 15 |
| Clinical characteristics | Cancer Type | Breast | 13 | 65 |
|  |  | Colorectal | 2 | 10 |
|  |  | Hodgkin Lymphoma | 3 | 15 |
|  |  | Melanoma | 1 | 5 |
|  |  | Thyroid | 1 | 5 |
|  | Disease Stage | Early | 8 | 40 |
|  |  | Late | 4 | 20 |
|  |  | Locally advanced | 4 | 20 |
|  |  | Unknown | 4 | 20 |
|  | Relationship Status | Living with partner | 8 | 40 |
|  |  | Married | 11 | 55 |
|  |  | Separated post-partum | 1 | 5 |
|  | Parity | Multiparous | 11 | 55 |
|  |  | Nulliparous | 9 | 45 |
| Birth outcomes | Birth Outcome | Full term | 4 | 23.5 |
|  |  | Overdue | 1 | 5.9 |
|  |  | Preterm | 12 | 70.6 |
|  | Mode of Delivery | Caesarean Section | 9 | 52.9 |
|  |  | Induced, vaginal | 6 | 35.3 |
|  |  | Spontaneous vaginal birth | 2 | 11.8 |

| **Supplement Table 3. Pregnancy-Associated Cancer: Key outcomes summary (N=20)** | | | |
| --- | --- | --- | --- |
| **Characteristic** | **Category** | **n** | **%** |
| Breastfeeding | Advised against by healthcare professional | 6 | 30 |
|  | Attempted only | 2 | 10 |
|  | Successfully breastfed | 7 | 35 |
|  | Unable/chose not to | 4 | 20 |
|  | Unknown | 1 | 5 |
| Treatment during pregnancy | Chemotherapy and Surgery | 1 | 5 |
|  | Chemotherapy only | 3 | 15 |
|  | Surgery only | 7 | 35 |
|  | Other treatment | 2 | 10 |
|  | No treatment | 7 | 35 |
| Treatment post-pregnancy | Chemotherapy only | 8 | 40 |
|  | Radiotherapy only | 3 | 15 |
|  | Hormone therapy only | 1 | 5 |
|  | Triple therapy (Chemotherapy, Radiotherapy and Surgery) | 1 | 5 |
|  | Other combination | 7 | 35 |
| Working status post diagnosis/treatment | Continued working | 7 | 35 |
|  | Maternity leave only | 3 | 15 |
|  | Stopped working | 4 | 20 |
|  | Sick leave only | 2 | 10 |
|  | Other | 1 | 5 |
|  | Unknown | 3 | 15 |

| **Table Supplement 4. Additional illustrative quotes for each theme** | |
| --- | --- |
| Theme | Illustrative quotes |
| ***Managing cancer with constrained choices: the weight of uncertainty*** | P1: [Describing having to wait for investigations/staging] Because they couldn’t give me a PET scan, they just gave me an MRI scan and kind of could see that there was a mass in my neck, which obviously was what we could feel, and in my chest also. So, they said it was stage 2. And then they had an MDT meeting  P3: I never had a mammogram until the morning after my [child] was born and that’s the only time that they could fully see everything, like all of the calcification. Up to that point, so between being 16-weeks pregnant and having my [child] at 35-weeks, I was told I’d need a lumpectomy, so mentally in my head that’s what I thought I was going to have, and it was only after they did the mammogram they said, “Actually, you are going to need a mastectomy on one side”. So that was a real shock, because I had maybe four months to get my head around a lumpectomy, I had six weeks to get my head around the fact that I needed to have a full mastectomy.  P9: I think one of the hardest things is they can’t (CT) scan you, so like you don’t know what stage you are at, so you are kind of preparing yourself for the worst.  P11: Well, obviously they can’t do the stage because they have to do the CT scan first, but I already knew that it had spread to the lymph nodes. That’s why I was more worried […] few weeks waiting for the scan because obviously it could have spread.  P13: [I was] HER2 positive and it needed immunotherapy, and you can’t have that whilst pregnant, so they needed to deliver [child] sooner so they could start me on a specific treatment.  P18: [After delivery] And then I had to go immediately for mammograms, blood tests and they start the process in terms of scans, because at that time all they knew was that I’d got a cancerous lump and that it was in my lymph nodes and my armpit as well. They didn’t know any more information than that, because I’d had no scans. |
| ***Ethical decision-making processes*** | P2: [After mastectomy] only then did I actually think oh my god I never actually asked for a second opinion, I really hope this was the right course of action because it’s happened now and I didn’t even think for a second to get a second opinion, which seems a bit crazy now I look back, but at the time it just didn’t really cross my mind.  P19: [There was] bit of a clash [between cancer and maternity teams] on deciding between whether to have an induction, because they wanted to bring the baby early, or have a C-section. My midwife argued for me with the chemotherapy it did make me tired and with an induction would I actually be able to give birth naturally because it’s just a long-lasting effect, even when the body has had a bit of a break, with the fatigue, and the length of labour can be unknown, it can go on for hours, obviously it can go very, very fast. So, she sort of argued for me a C-section would be best. The consultants were a bit concerned because obviously infection control risk again with it being surgery, but it was agreed that I could have a C-section  P20: They wanted it so I could begin treatment. But I'm very angry about it, to be honest, because [child] was born early, [child] then had trouble breathing and was in NICU. So, they didn’t do anything with me until […] [month], when I had an MRI scan. And I thought, ‘Why couldn’t that have just […] [child] could have stayed in there. [child] didn’t need to come out that quickly when they didn’t even have an MRI scan booked. I had to wait for the appointment. So, I don’t understand why they couldn’t have left [child] there longer. |
| **Balancing cancer and its treatment with pregnancy and family life** | P10: So, going up to visit the baby (on ICU) was really hard, because I had to be taken in a wheelchair by someone else. I couldn’t get there myself, and things like that. I was finding it hard to express the milk. I wasn’t really eating much, so the milk wasn’t really coming, and all these sorts of things. I probably worried about the baby more than I was really thinking about myself. I was just worried that I wasn’t going to visit enough, and everyone would think I was an awful mother, because I was never up there. All the other mums would sit by their incubator all day, but I would go there for about ten minutes, and I just couldn’t sit there anymore, because I was so ill.  P13: So then after I had [child],I had a week off and then started back on chemotherapy again for another nine weeks, so that was quite gruelling.  P18: So, [after] my first operation and [then] my mastectomy, I wasn’t able to pick [child] up for six weeks, which was emotionally difficult, because you’re there; It was hard at times […] emotionally difficult, because you’re there […] And it was really, really tough. You get used to having [child] placed on your lap and when [child] is three, four months old that’s fine. But when [child] was then getting on for a year old, when I had the mastectomy. |
| ***Work disruption and financial strain*** | P5: Staying away from home and children [during radiotherapy] that was another real mental headache of what am I going to do? Where am I going to go? How am I going to pay for it? All that sort of thing. So, I was quite lucky that the hospital charity funded some accommodation for me, which was really good. Because it was more expense that I didn’t need, to be frank.  P14: I was due to start a new job in the [month], and [my oncologist] said, “If there’s anything you can do not to start that job then don’t ,” and oncologist was quite right because I’d have been starting it less than two weeks after diagnosis. I don’t think mentally I was in the right frame of mind to take on a new job managing people […] If I had been in work at the time, I don’t think I’ve had ever been at work because I was just so busy with appointments. Even when they were on the telephone and things, it’s still taking up a lot of your time. |
| ***Emotional impact of diagnosis and treatment*** | P1: I was really, really frightened. It was terrifying really.  P13: Shock and fear were the main two [feelings]  P9: I think it was shock. [pause] How? [pause] That you’re going to die. For the first couple of weeks.  P15: I kept breaking down into tears anytime anyone talked about breastfeeding or every time I brought up breastfeeding […] it was just heart-breaking |
| **Coping, adjustment and support** | P2: Yeah, so the consultant was very good. And the fact that he phoned me back immediately when I had questioned whether I should be going into hospital or not, I thought that was very good. My breast care nurse, who I was given to sort of look after me, she was amazing around the time of diagnosis and leading up to my surgery, she was great, and she would phone quite regularly to give me updates or not. Even if I was expecting an update but they didn’t have an update, she would call me anyway, and she was quite hands on, up to the operation, and my midwife was amazing, and in fact my midwife came with me because my husband wasn’t even allowed in with me when I had the mastectomy (due to Covid -19 visitor restrictions), he dropped me off and then went home, but then my midwife did come with me.  P11: Obviously it’s not a nice thing but it is nice to have similar people to connect with because obviously you go to the local charity support group and they are all older. Which is still nice, but you are sort of looked at to be the outsider because you are young. Not that people [don’t have] like sympathy but people obviously are a lot older, they look at you a type of way, look at her, she’s only young. But these people, you connect with (through specialist charity for women diagnosed with cancer during pregnancy), they are all in the same boat, so I find it very helpful. Even just advice. Mums going through the treatment that they are going through, and I don’t have anyone who I can relate to apart from that group. I’d say, if anything, probably more for the positive, I think it’s just bringing me closer to people, like friends, literally everyone, all my friends, family, my partner especially, he’s been my biggest support ever through it all…everyone sort of pulls together in these situations to help us as a family, the kids  P12: [Describing living in survivorship with her perceived “delayed” diagnosis] What would I have needed to do to make you have dealt with this in a more efficient manner? Because as we know, the earlier this is dealt with, the less chance it has of coming back. And that stands now. I still think I have a higher risk of recurrence now because it was left so long […] I have the constant fear that it’s in my bloodstream, so what will it take for it to manifest somewhere? It happens quite frequently […] So I feel like now, if I am to have a recurrence, I would never know, and that’s just as bad. I would never know, if I would have been seen earlier, if that would that have prevented that. I think psychologically that’s difficult to deal with. |

| **Supplement Table 5. Diagnosis of cancer during pregnancy: preliminary recommendations for the extended multidisciplinary teams (MDTs)** | |
| --- | --- |
| **Area** | **Implications for cancer, maternity and wider health and social care professions** |
| **Birth and parenting experience** | - Acknowledge and validate the pregnancy, recognising its emotional significance alongside the cancer diagnosis. - Where clinically appropriate, support as normal a birth experience as possible while balancing maternal and foetal risks. - Initiative early, open conversations about individual values and plans (e.g. birth preferences, breastfeeding, childcare) as part of treatment planning. - Ask directly about hopes or fears related to the birth or postpartum period and document these to ensure continuity across the extended MDT. |
| **Decision-Making Support** | - Provide clear, comprehensive, and unbiased information about treatment options and potential trade-offs. - Support shared decision-making processes individual values and priorities. - Avoid pressuring patients into decisions that conflict with personal values. - Explicitly acknowledge the complexity of dual cancer-pregnancy decisions. |
| **Psychological Support** | - Adopt trauma-informed care principles, including emotional safety, trust, collaboration, empowerment, and choice. - Offer/refer to evidence-based psychological assessment/therapies (e.g. CBT, ACT, trauma-focused CBT) matched to individual needs and preferences. - Where available, provide access or signpost to ongoing psychosocial support rather than single time-point interventions, recognising variable readiness over time. - Normalise delayed psychological processing following period of ‘survival mode’ during acute treatment and early parenting. - Recognise common themes such as maternal guilt, identity disruption and fear of recurrence, and integrate psychological approaches that address self-compassion, values and cognitive distortions. |
| **Care Coordination** | - Establish integrated multidisciplinary care spanning oncology, maternity, and relevant supportive services. - Improve communication and information-sharing between specialties to reduce fragmentation. - Where possible, designate a care coordinator or key contact to reduce navigation burden. - Develop and implement clear local protocols for pregnancy-associated cancer care. |
| **Financial Support** | - Provide early information about financial assistance, employment rights, and benefits where available. - Where possible, connect women with social workers or support staff familiar with pregnancy-associated cancer. - Signpost to organisations that can assist with workplace accommodations and advocacy. - Proactively address financial concerns as part of survivorship planning. |
| **Long-term Survivorship** | - Develop a survivorship care plan addressing physical follow-up, mental health, fertility, and parenting needs, and ensure this is shared with relevant services (e.g. primary care, maternity/fertility, health visiting/community nursing). - Consider screening for delayed emotional responses (e.g. decisional regret, anxiety, adjustment difficulties), particularly during the first 1-2 years post-treatment. - Ask directly about ongoing maternal responsibilities, fatigue and functional recovery, and offer appropriate referrals (e.g. occupational therapy, parenting and social support, perinatal services). - Monitor for signs of trauma-related distress (e.g. hypervigilance, intrusive thoughts, avoidance) and refer to appropriate psychological services. - Use follow-up appointments to revisit evolving issues such as return to work, parenting challenges, fertility, or future pregnancy planning. |
| **Information & Resources** | - Provide accurate, tailored written information about pregnancy-associated cancer at diagnosis, in accessible formats where needed. - Signpost to pregnancy-associated cancer-specific resources and education materials. - Provide details of relevant peer or support groups, where available. - Ensure clear pathways for accessing psychological care, including support for delayed needs in survivorship |
|  |  |

| **Supplement Table 6. Diagnosis of cancer during pregnancy: preliminary recommendations/implications for survivors/families** | |
| --- | --- |
| **Area** | **Implications for survivors and families** |
| **Birth and parenting experience** | - Communicate with HCP about your hopes and expectations for birth experience and parenting, even if they feel “small” compared to medical treatment. - Ask your team about how treatments may impact your preferred birth plan (e.g. timing, place of birth, use of pain relief, breastfeeding). - Clarify breastfeeding goals early and request to speak to a lactation consultant if needed. Ask the team to explain medication adjustments or treatment pauses. - Make a flexible plan for childcare support, especially during admissions, treatment cycles or appointments. Where possible, try to involve trusted family/friends ahead of time. - If you feel misunderstood or overwhelmed, ask for a patient advocate, maternity liaison, or communication support. You have the right to feel heard. - Acknowledge that your birth experience may need to adapt for safety, but know that meaningful, empowering experiences are still possible with support. |
| **Decision-Making Support** | - Acknowledge that it’s okay to feel overwhelmed when balancing pregnancy and cancer-related decisions. - Write down your personal values and preferences early in treatment planning and share this with your care team - If you feel rushed or conflicted, ask your team to “pause and explain” options clearly. You are entitled to understand all medically reasonable choices. - Request decision-making aids or bring a trusted support person to help review complex choices. - Seek a second opinion if you’re uncertain or feel your concerns are being dismissed. This is a normal and accepted part of care. - Advocate for decision-making time when medically feasible, e.g. agree to schedule a follow-up appointment specifically for decision review, to avoid snap decisions during high-stress moments. - Continue to journal or write down your questions, preferences and evolving concerns to help you guide conversations with your team. |
| **Psychological Support** | - Recognise that "survival mode" during treatment may limit capacity for emotional processing. Seek psychological support if needed and when ready. This varies but may be potentially months or years post-treatment - Know that it's normal to need emotional support months or even years after treatment, especially once immediate medical concerns subside. - If experiencing distress, guilt, or low mood, consider seeking help from a CBT therapist (to address unhelpful thought patterns) or ACT therapist (to explore values, accept difficult emotions, and build psychological flexibility). - Feelings of guilt, regret, or loss around pregnancy or parenting experiences are common and these are treatable concerns, not personal failings. - Consider connecting with peer support groups (e.g. in the UK Mummy’s Star, in the US Hope for Two), which can provide validation and reduce isolation. |
| **Care Coordination** | - Expect to interact with multiple care teams (e.g. oncology, maternity, surgery, mental health). Keep a personal care log with appointment summaries, key contacts, and questions. - Identify and write down the name and contact information of your main care coordinator or lead clinician. Ask your team to clarify who this is. - Bring a trusted person to appointments if you need help tracking complex information. - If you're feeling overwhelmed or uncertain who to contact, ask directly for care coordination support (e.g. specialist nurse, social worker). |
| **Financial Support** | - Ask your care team early on about financial support options. Some hospitals have dedicated social workers or welfare advisers, or they can put you in touch with external support. - Track treatment-related costs (e.g. travel, childcare, lost income) to support welfare/benefit claims or workplace discussions. - Familiarise yourself with employment protections for pregnancy and cancer (e.g. time off, adjustments, sick leave). Consider speaking with a workplace union or employment/legal advisor if needed. - If possible, make a flexible financial plan for the months following treatment, especially if you’re balancing caregiving responsibilities or considering changes in work. |
| **Long-term Survivorship** | - Understand that emotional and physical recovery often extends beyond active treatment. Delayed emotional responses, including grief or regret, are common and valid. - Consider keeping a health and wellbeing journal to track physical symptoms, mood, parenting challenges, and questions for follow-up care. - Ask for a survivorship care plan, especially if transitioning back to primary care. - Prepare for potential delayed emotional responses - Develop strategies for managing ongoing caregiving responsibilities - Where available/possible, you may need to maintain connections with survivorship care team for extended periods |
| **Information & Resources** | - Prioritise reliable, targeted resources. Look for information from cancer charities or clinical sources specific to pregnancy-associated cancer - Consider joining peer support groups or online forums tailored to people navigating both cancer and parenthood. It can be good to connect with other people who have similar experiences but remember everyone’s journey is different. - Ask your team or a trusted source for guidance on parenting decisions post-treatment (e.g. breastfeeding, future fertility, explaining your cancer experience to your child). - Bookmark your list of recommended websites or services so you can return to trusted information when needed. - Learn about long-term monitoring needs from your team, including fertility, mental health, and risk of recurrence. Ask for a plain-language explanation. |
|  |  |
